# Set-shifting and inhibition interplay affect the rule-matching bias occurrence during conditional reasoning task

**DOI:** 10.25122/jml-2021-0215

**Published:** 2022-06

**Authors:** Seyyedeh Fatemeh Seyyed Hashemi, Reza Khosrowabadi, Mohsen Karimi

**Affiliations:** 1 Department of Cognitive Psychology, Institute for Cognitive Science Studies, Tehran, Iran; 2 Department of Cognitive Modeling, Institute for Cognitive and Brain Science, Shahid Beheshti University, Tehran, Iran.; 3 Department of Computer Engineering, Institute for Artificial Intelligence and Robotics, Amirkabir University of Technology, Tehran, Iran

**Keywords:** conditional reasoning, rule-matching bias, set-shifting, inhibition

## Abstract

The rule-matching bias is a common error during conditional reasoning tasks, which refers to a tendency to match responses with the lexical context in the conditional rule and leads to incorrect responses. Conditional reasoning is one of the higher-level cognitive abilities affected by many cognitive skills. We aimed to determine whether inhibition and set-shifting skills with rule-matching bias occurrence could be related and, if so, to what quantitative, at a statistically significant level. A total of 30 healthy university students aged 18 to 30 participated in this study. We used the Wason's Selection Task (WST) to measure conditional reasoning and investigated their inhibition and set-shifting skills with the Stroop and Wisconsin Card Sorting Test, respectively. Results showed a significant positive correlation between the number of correct responses to the Stroop test and the Wason Selection Card Test (p=0.614). There was a positive correlation between the number of correct responses to the Wisconsin Card Sorting Test and the Wason Selection Card Test (p=0.423). Participants with higher inhibition and set-shifting abilities showed better performance in the conditional reasoning test and lower rule-matching bias errors.

## INTRODUCTION

Reasoning ability is central to human cognition [1]. Understanding social norms and rules, making scientific deductions [2], and even many everyday life decisions like comparing two routes to reach a single destination and choosing one are made possible by reason [3]. Reasoning involves a multistep examination of the received information and drawing relationships between them to provide a conclusion [4]. Reasoning comes in many forms, including inductive and deductive reasoning [5]. In inductive reasoning, a general conclusion is drawn based on examining specific information. Hence, inductive reasoning can be considered a "bottom-up" process in which finding similarities between propositions plays a key role, and there is no guarantee that the conclusion is correct [4]. For example, every swan I have seen so far has been white (specific information); this swan is also white; therefore, all swans are white (general inference). Instead, in deductive reasoning, specific conclusions are drawn based on a general rule, and the correctness of the conclusion is guaranteed. Hence, deductive reasoning is considered a "top-down" approach. For example, all human beings are mortal (a general fact). Socrates is a human being. So, Socrates is mortal (particular result) [6].

Conditional reasoning, known as "if... then..." is a kind of deductive reasoning in which inferences and conclusions are formed based on comparing pre-existing information with new information. Understanding causation and predicting possible outcomes is possible through conditional reasoning [7]. A set of cognitive skills is needed in the conditional reasoning process to create a valid logical conclusion and, ultimately, the correct answer. Due to limited mental resources, the process of reasoning does not always go as it should, and sometimes reasoning becomes erroneous [8]. For example, the logical inference is sometimes erroneous due to the rule-matching bias that refers to a tendency to adapt the responses to the lexical content presented in the conditional statements [9]. Rule-matching bias is a vital phenomenon in conditional reasoning, which is almost entirely dependent on implicit negation. If the participants are satisfied with the familiar option as the correct response without deduction, the conclusion would be considered invalid due to the rule-matching bias. Behind the expressions, there are logical conditions that their understanding requires receiving and processing information that is not explicitly stated. Therefore, biases will occur even when participants have the analytical capability in case of failure to obtain the relevant information [9].

In order to avoid the rule-matching bias occurrence, it is necessary to update the information to enable focused processing [10]. During the information update process, irrelevant information is actively removed and replaced with relevant information [11]. Therefore, cognitive skills such as attention, working memory, and functional connectivity between them are crucial [7]. Indeed, attention skills allow the conscious selection of information [12], and working memory enables information to be retrieved, stored, and manipulated during the reasoning process [13]. Furthermore, creating a connection between attention and working memory requires cognitive flexibility [14], which refers to the ability to update behaviors in the face of a changing environment [15].

Set-shifting is considered one of the internal mechanisms of cognitive flexibility that enables individuals to change rapidly from one criterion or rule to another [16]. Set-shifting makes it possible to self-update, exchange, and store information in working memory [17]. First, the individual needs to select the related pieces of information – proper attention directing – [18], which means that the reasoning process requires inhibition skills to withhold unwanted responses and redirect attention to the target information. Second, the information under review must be updated via set-shifting skills because removing outdated information from working memory allows for replacing irrelevant information with updated, relevant information [19]. Examining the effect of cognitive flexibility on the decision-making process in uncertain situations showed that people with higher cognitive flexibility performed better in working memory, inference tasks, and probabilistic learning tasks such as the Iowa gambling task, which includes making decisions under uncertainty [20].

Conversely, people with less flexibility (for example, people with obsessive-compulsive disorder) have a lower ability to reason correctly, which would result in an inability to appropriately direct attention and inhibit irrelevant information or disruption in updating information via set-shifting [21]. In addition, findings show that people with higher cognitive flexibility skills need less time to learn rules and are faster in processing the meaning of received feedback [22]. Thus, cognitive flexibility skill facilitates people's access to the right strategy to deal with the problem ahead by providing optimal mental resources quickly.

Although several studies have examined the role of working memory on conditioned reasoning performance [23–25], inhibition and set-shifting skills for making conditional inferences based on difficulty level breakdown remain unclear. During conditional reasoning, when a conditional statement as "if p then q" is presented, four conditional modes are expected [26]: 1) p, therefore, q; 2) not-q therefore not-p; 3) not-p therefore not-q; and 4) q, therefore, p. Participants were told that a total of four cards (p, q, not-p, and not-q) are presented [27], and each card has two sides; there is a number on one side and a letter on the other. Participants were asked to select the minimum number of cards to turn over to test the validity of the conditional statement. In the first inference, "p, therefore, q", if the card with the symbol "p" is turned over and the symbol "q" is on the reverse side, the conditional statement is confirmed. If any symbol other than q (=not-q) is on the reverse side, the conditional statement is disconfirmed. So, the card containing the symbol "p" is able to validate the conditional statement. In the second inference, "not-q, therefore, not-p", if the card with the symbol "not-q" is turned over and the symbol "p" is on the reverse side, the conditional statement is disconfirmed because it is against the conditional rule which was presented at first. Whereas, if the symbol "not-p" is on the reverse side, it would not confirm or disconfirm the validity of the conditional statement because it has nothing to do with the conditional statement rule. In the third inference, "not-p therefore not-q", if the card with the symbol "not-p" is turned over, regardless of whatever is on the reverse side of the card, the conditional statement is neither confirmed nor disconfirmed; because there is no specific explanation about this inference in the conditional statement. So, the card with the symbol "not-p" is not able to validate the conditional statement. In the fourth inference, "q therefore p", if the card with the symbol "q" is turned over and the symbol "p" or "not-p" is on the reverse side, the conditional statement is neither confirmed nor disconfirmed; because the conditional statement is not bidirectional [18]. As a result, the card containing the symbol "q" cannot validate the conditional statement. So, the first two inferences are valid, and the third and fourth inferences are invalid [28]. According to previous studies, it is not difficult for healthy people with natural intelligence to understand the logic of the first and third inferences. Still, the logic behind the second and fourth inferences is challenging to understand, so there is a potential for rule-matching bias when responding to them. In addition, studies show that only 5 to 7 percent of very able participants can provide correct responses and accurate explanations about the logic behind questions [3]. Given that conditional reasoning allows the conclusion of a hypothesis in any field, including everyday issues and scientific issues [2], identifying the factors affecting it seems necessary.

This study aimed to investigate the relationship between inhibition skills and set-shifting skills on the incidence of rule-matching bias during performing conditional reasoning tasks. Conditional reasoning, set-shifting skill, and inhibition skill in participants were assessed using the letter and number versions of the Wason Card Selection Test, the Wisconsin Card Sorting Test, and the Stroop Test. A version consisting of the letters and numbers of the Wason's Selection Card test was used to eliminate the effect of meaning, syntactic and content processing, and the possibility of other biases such as belief bias. Based on the findings showing that the work memory performance and attention types are interdependent, many cognitive skills are needed to establish and maintain this relationship in the long run [29, 30]. This connection is established by the internal mechanisms of cognitive flexibility skills, which provide a suitable platform for thinking. Accordingly, in this study, we hypothesized that individuals with higher set-shifting and inhibition skills would select relevant information and keep them present in working memory to provide a valid response away from biases, even in the limited time.

## MATERIAL AND METHODS

### Participants

Thirty-two students participated in the experiment. Data from two participants were excluded from the study due to responding too quickly or misunderstanding the instructions. A total of 30 participants (17 women) aged 18 to 30 years (M=25.7, SD=2.5) were included for further analysis. All were right-handed, had normal or correct-to-normal vision, had no MRI counter-indications, and were absorbed through advertising. Exclusion criteria included the history of illness (mental, neurological, and medical), head injury, anesthesia, lactation, pregnancy, and alcohol or mixed budget. After training, all participants gave their informed written consent and were paid to participate.

### Procedure and assessment

This study was a cross-sectional study and part of an fMRI (Functional Magnetic Resonance Imaging) study. In this study, participants' behavioral data were explored and analyzed separately during the Wisconsin Card Sorting Test, Stroop Test, and Wason Selection Test. Participants' general health was examined and confirmed by a physician before the experiment. After signing the consent form, participants completed a demographic information questionnaire. Participants were then trained separately on how to complete the Wason's Selection Card and Stroop tests. Participants underwent a training program with explanations of the analytical knowledge involved in testing conditional expressions, followed by a personal training session on performing a computer task. The participants practiced each of the five types of Wason test symbol probes until their performance reached at least 60% accuracy. The training program helped to minimize the effect of individual differences. Participants were also taught to use the answer box buttons to answer the Stroop test questions. The Wason and Stroop tests were randomly assigned to participants on an MRI scanner, and it took approximately 22 minutes for the participants to complete both tasks. The Wisconsin Card Sorting Test was performed outside the MRI scanner.

### Measures

#### Conditional reasoning

**Figure 1:**
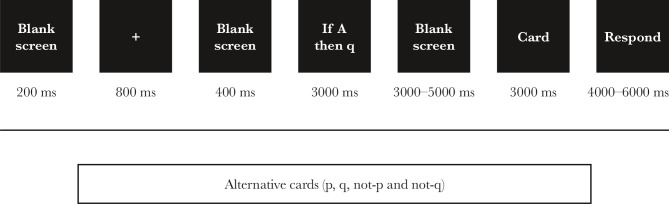
Schedule presentation of the test pattern of the Wason Selection Card Test.

The standard version of Wason's Selection (WST) [31] for fMRI was programmed using the Psych toolbox in Matlab software. Figure 1 shows the design of the Wason Selection Test. A total of 9 conditional sentences (45 questions) were distributed in two blocks, with the first block containing 25 and the second containing 20 experiments. Each conditional expression consisted of four parts: 1) a general expression, 2) a conditional expression, 3) four substitute cards, and 4) a control condition containing an irrelevant mathematical symbol such as "٪", which was entirely irrelevant for the conditional proposition. To run the task inside the scanner, control condition symbols that did not exist in the original version were added to the standard version of Wason's Selection. The general statement was the same for all experiments and was displayed only once. The four alternate cards consisted of two single-digit numbers and two non-compound letters. So, each trial period started with a condition statement followed by the alternative cards as p (letter E), q (number 4), not-p (letter other than E such as G), not-q (number other than four such as 7), and an irrelevant card was presented as a control. Participants were asked to indicate which card could potentially disconfirm the conditional statement. As previously explained, only two cards containing "p" and "not-q" could disconfirm the condition statement, so the "q", "not-p", and control card containing a mathematical symbol such as "٪" could not confirm the condition statement.

As can be seen in Figure 1, each test period started with a blank page for 200 milliseconds; immediately after presenting the cue sign (+) for 600 milliseconds, a blank page was displayed for 4000 milliseconds, and then the rule of logic (conditional expression) was displayed for 3000 milliseconds and created the relationship between the variables (for example, "if A then 7"). After presenting the conditional expression, a blank screen was displayed at a random time interval of 3000–5000 milliseconds, and immediately, the probe symbol was presented for 3000 milliseconds. In providing a probe symbol, each card contained either a number or a letter (for example, one of five possible modes: "p", "q", "not-p", "not-q") or a stimulus other than the alphabetical symbol (for example, "٪" was considered as a control). The stimuli were predicted to be seen through a mirror on the coil in the scanner. At the end of the stimulus presentation, two answer options (*i.e*., "Yes" or "No") were displayed for 4000–6000 milliseconds, and participants were instructed to answer as quickly as they could. To answer, participants had to choose "Yes" or "No", depending on the card provided. For example, when the card was presented with the letter "E" (=p), the correct answer was "yes". Responses were recorded through two MR-compatible response boxes that worked with thumb pressure. Response "Yes" was recorded by pressing the left thumb, and response "No" was registered by pressing the right thumb.

#### Set-shifting

The Wisconsin Card Sorting Test (WCST) [32] was used to measure participants' cognitive flexibility as an indicator of set-shifting performance. Four cards incorporating three stimulus parameters (color, shape, and number of objects) were presented to the participants, and they were instructed to match a new card to the "correct" old cards. Participants guessed which old card matched the new card and got feedback, "correct" or "incorrect". The participants gradually learned the rule for "correct" responses. The criterion for "correct" old card kept changing over the blocks of trials. In normal performance, participants can learn to switch the criterion for a new block of trials. Set-shifting skill is reflected by fewer errors. The WCST is not a time-limited test, but it approximately lasts 20–30 minutes and ends after creating six categories or after the presentation of all 64 cards is completed.

#### Inhibition

The cognitive inhibition skill was examined using a color-word Stroop task [33]. In this task, participants were instructed to name the color that the word is printed in, while ignoring the meaning of the word. In compatible conditions, the color words were written with the same color ink (*i.e*., BLUE was written with the blue ink color) whereas, in incompatible conditions, the color words were written with different color ink (*i.e*., BLUE is written with the red ink color). Obviously, the incompatible condition is harder as it needs self-control and inhibition. In the present study, we used an event-related color-word Stroop task in which four target color words (*e.g*., red, green, yellow, blue) were presented in the compatible or incompatible trials. This task comprised two parts (36 trials) in which each trial was presented for 2000–3000 ms, and the blank screen was presented between trials for 2±1 s as the interval. Participants had to name the color of ink that the word was printed in as soon as possible. Participants' responses were submitted by pressing the buttons (*i.e*. green=left thumb, blue=left index, red=right thumb, yellow=right index) of two response boxes.

### Analysis strategy

Statistical analysis was performed using SPSS 22.0. For descriptive statistics, mean and standard deviation were used. We conducted data analysis based on correct and incorrect answers, and the missing responses were considered the inability to provide a response due to time constraints. So based on the purpose of the present study, we did not include missing responses for analysis. An approach called Linear integrated speed-accuracy scores (LISAS) combined the proportion of errors and the reaction times into a single score which led to the study of the cognitive tasks in a more reliable and parsimonious way. The LISAS score based on response time (RT) and incorrect response ratio (EP) for the j person in the study was shown as follows:


$$Lisas=RT_{j}+\frac{S_{RT}}{S_{EP}}\times EP_{j}$$

The S_RT_ is the standard deviation of individuals' response time, and S_EP_ is the standard deviation of incorrect responses in a study based on the results of all individuals in the study.

In normally distributed groups, the result was presented with a mean and 95% confidence interval. We conducted a Pearson correlation test between LISAS Wason's and LISAS Stroop and a one-way ANOVA analysis of variance between LISAS Wason's and Wisconsin. The LISAS Stroop and Wisconsin's performance were considered indicators of inhibition and set-shifting skills, respectively. The level of significance was set as α=0.05.

## RESULTS

9 Ph.D., 13 masters, and 8 undergraduate university students participated. The hypothesis of normality of the data based on the Shaphiro Wilk test was not rejected at a significance level of 0.05; as a result, the data had a normal distribution. The results of the descriptive statistics are shown in Table 1.

**Table 1:** Descriptive statistics of Wisconsin, Wason, and Stroop tests.

Measures	Variables	Mean	SD	CI
**Wason's Selection Card**	RT	4.767	0.114	4.762, 4.772
	CR	26.57	6.725	26.219, 27.921
	FR	7.070	5.521	6.71, 7.43
**Stroop**	RT	1.617	0.300	1.414, 1.820
	CR	28.330	3.315	27.881, 28.119
	FR	7.670	3.152	7.302, 8.038
**Wisconsin Card Sorting**	Category number	4.370	1.033	3.908, 4.732
	FR	9.830	4.778	9.121, 10.533
	P	1.570	3.256	1.480, 1.980

RT – Reaction Time; CR – Correct Response; FR – False Response; p – perseverate errors; CI – Confidence Interval.

The reaction time (in seconds), the number of correct and incorrect responses, plus the combined Lisas factor, were the variables measured for the Wason's Selection Card and Stroop tests. In the Wisconsin Cards Test, the number of categories made and incorrect responses, and perseveration errors were investigated. The confidence intervals of reaction times and correct and false responses in Wason's and Stroop tests did not overlap. The confidence interval of the number of categories made, false response, and perseverate errors of the Wisconsin test did not overlap, as well. The mean and standard deviation of correct responses in Wason's test with 95% CI=[26.219, 27.921] was M=26.57, SD=6.725. The mean score shows that more than 50% of Wason's test questions were answered correctly. The mean and standard deviation of correct responses in Stroop test with 95% CI=[27.881, 28.119] was M=28.330, SD=3.315. The mean score shows that more than 65% of the Stroop test questions were answered correctly. The mean and standard deviation of reaction time in Wason's test with 95% CI=[4.762, 4.772] was M=4.767, SD=0.114 and in Stroop test with 95% CI=[1.414, 1.820] was M=1.617, SD=0.3. The mean and standard deviation of the number of categories made in the Wisconsin test with 95% CI=[3.908, 4.732] was M=4.370, SD=1.03. The mean indicates that the classification in the Wisconsin test is more than 60% correct. The descriptive statistics of Lisas of Wason's Selection Card Test and Stroop Test are shown in Table 2.

**Table 2:** Descriptive statics of Lisas Wason and Lisas Stroop.

Measures	Mean	SD
**LISAS Wason**	4.91	0.14
**LISAS Stroop**	2.31	0.39

LISAS – a combined index to measure variance.

The combined index of LISAS was made for Wason's test with M=4.91, SD=0.14, and for Stroop test with M=2.31, SD=0.39. According to the linear correlation conducted by the Pearson correlation test shown in Table 3, LISAS Wason was positively correlated with LISAS Stroop (r=0.614, p=0.036, d=1.5 at a significant level of 0.05 and 95% CI=[0.339, 1.091]) with an increase in LISAS Wason resulting in a corresponding increase in LISAS Stroop. As the error in the Stroop test increases, so does the error in the Wason test, and higher performance levels on the Stroop test led to higher performance levels on the Wason test. A positive correlation (r=0.423, p=0.045, d=0.9, at the significant level of 0.05 and 95% CI=[0.075, 0.827]) was observed between the performance of the Wisconsin card sorting test and the Wason's test. Participants with better performance in the Wisconsin test showed better performance in Wason's test. A marginally positive correlation r=0.249, p=0.051, d=0.5 was observed between Wisconsin test and Stroop test performance at a significant level of 0.05 and 95% CI=[-0.142, 0.61] which did not reach statistical significance because of null trials. Based on Pearson correlational results, the participants with more inhibition ability in the Stroop test and more set-shifting ability in the Wisconsin test also performed more accurately on the Wason's test.

**Table 3:** The correlation between Wason's Selection Card, Wisconsin Card Sorting, and Stroop tests.

Measures	Variables	1	2	3
**(1) LISAS Wason**	r	-	-	-
	p-value			
	CI			
**(2) LISAS Stroop**	r	0.614	-	-
	p-value	0.036		
	CI	0.339–1.091		
**(3) Wisconsin**	r	0.423	0.249	-
	p-value	0.045	0.051	
	CI	0.075–0.827	-0.142–0.61	

r – Correlation; CI – confidence Interval; p-value ˂0.05.

## DISCUSSION

In the current study, we examined the role of cognitive flexibility in order to learn how conditional reasoning relies on executive functions. In particular, the possible effect of set-shifting and inhibition skills on reducing the incidence of rule-matching bias during the conditional reasoning task was explored. We hypothesized that participants with higher inhibition and set-shifting skills have better reasoning ability and are less likely to make mistakes. Our results showed that the participant's performance in the Wason Selection Test was significantly related to the Stroop Test and the Wisconsin Card Sorting Test. A positive correlation was seen between the LISAS Wason and the LISAS Stroop indexes. There was also a marginally positive correlation between the LISAS Wason index and the level of performance in the Wisconsin Card Sorting Test. The implications of these findings, as well as the limitations and strengths, are discussed as follows.

Although there was a marginally positive correlation between the performance of Wisconsin and Stroop tests, we cannot draw a strong conclusion because of the null results. A proper possible explanation for the presence of this relationship is that while performing the WSCT test, the inhibition skill is required to shift from one sorting rule to another, like from color to shape. Using the inhibition skill helps suppress the attention from the previous rule and redirect to the new one [34]. Studying children's performance in the WSCT test revealed that the low-level performance results from the late understanding of changes in sorting rule and the lack of rapid shifting and switching ability [35]. Closer inspection of the WCST test structure reveals that multiple additional cognitive processes, including category formation, set maintenance, working memory, and especially inhibition skill, are needed. So, even highly flexible individuals may commit many errors by not redirecting their attention and updating the wanted information in working memory [16, 34]. These findings revealed that the set-shifting and inhibition skills are independent but still correlated with one another [36].

Based on the present study results, higher inhibition and set-shifting skills reduce the incidence of rule-matching bias, leading to superior performance on logical analogy-based reasoning. These results are consistent with a study in which, by examining brain activity with the ERP method, it was determined that N2 activity was seen when responding correctly to the cards containing the symbol "not-q". The N2 is an endogenous event-related brain potential component related to inhibition and cognitive control that implies processing conflict information [18]. Therefore, answering these questions requires cognitive control and proper use of mental resources. In particular, during Wason's Selection Test, responding to the cards containing the symbols "q" and "not-q" that examine the validity of the inferences "q therefore p" and "not-q therefore not-p" respectively are more challenging and prone to the occurrence of rule-matching biases [37]. If the main components of the test are summarized, like the Wason's Selection Test, there is a strong tendency to match the content of the think with the obvious signs of the conditional proposition [28]. There is a notable tendency to pay over-attention to positive symbols (the cards containing p and q) and select them as the correct cards for testing the validity of conditional statements, which hinders the process of correct reasoning while performing Wason's Selection test [9, 38]. These findings support the present study's hypothesis, which stated that understanding the logic underlying the questions containing the symbol "q" is challenging, leading to failure in preventing extreme attention to the explicit sign in the conditional statements. In response to the question of whether the card with the symbol "q" would validate the conditional statement or not, participants who are trapped in biases choose the answer "Yes", based on the presence of this sign in the conditional statement, while the correct answer is "No".

Target information retrieval and updating in negative conditional statements (like not-q) compared to positive conditional statements (like p and q) sound more complex, leading to cognitive conflicts [39]. Providing the correct response and dealing with cognitive conflict strongly depend on examining the implicit logic behind the question [26]. Considering two possible conditions and deciding by adapting them to the conditional inference and meaning derived from the conditional statement is crucial to correct responding [26, 40]. The present findings showed that participants with higher set-shifting skills are less likely to be prone to the rule-matching bias, which means that they have a higher ability to update relevant information in working memory. The set-shifting, a multifaceted skill responsible for keeping task sets present in working memory [41], provides instant access to all related information and makes it possible to devote more attention to comparing them with the information presented in the conditional statement [14]. The results are broadly in line with the findings; using fMRI showed that when responding to the not-q question modes, more stable and extended processing is required than in other modes. A more comprehensive range of brain activity is observed, which means that the card containing not-q has a significant load on the working memory capacity [18], so correct responding requires strategy choosing and paying more sustained attention. It is particularly noteworthy that choosing a strategy needs to integrate proper attention directing and inhibition skills [41, 42]. Higher inhibition ability would help participants structure their thinking [43, 44] and use the analogy-based strategy while doing reasoning tasks.

Previous studies showed that for practical reasoning without any biases, a large working memory capacity is needed to suppress the heuristic reasoning strategy and replace it with the analytical thinking strategy [23]. The heuristic reasoning strategy is based on behavioral economics and often leads to failure in logical thinking. Consider this example: A participant is given the conditional statement "if E then 4", which is equivalent to "if p then q", then he/she is asked to respond whether the card contains the number "7" that equals "not-q" can disconfirm the conditional statement. Two modes may occur when the card is turned over; one is when the letter "E" that equals the "p" is on the reverse side of the card, or if a letter other than "E", like "G" that equals the "not-p". If the card containing the number "7" is turned over, and the letter "G" that equals the "not-p" is on the reverse side, then nothing happens against the conditional statement rule. So, the conditional inference "not-p therefore not-q" can test the validity of the conditional statement. Whereas, if the card containing the number "7" is turned over and the letter "E" that equals the "p" is on the reverse side, then it is against the conditional statement because based on the conditional statement presented, it is expected that behind the card containing the letter "E", it is the number "4" and not the number "7"; otherwise, the conditional statement is disconfirmed.

Based on the present results, the relationship between set-shifting skill and conditional reasoning was less than the relationship between inhibition skill and conditional reasoning. A possible explanation for this finding is two features of the Wisconsin Test: 1) receiving feedback and 2) no response time limitation. During the WCST test, if the participants respond wrongly, they can correct the response by receiving feedback. In comparison, there is no help to guide the way of thinking and no opportunity to correct the responses in the Stroop Test. On the other hand, the WCST has no response time limitation, whereas during the Stroop Test responding within the specified time frame is required. It is important to note that responding in a limited time may increase errors and performance deficits [45]. The participants' overall performance in the Wisconsin test was relatively high, with perseverate error rate of almost zero and a minimum of three categories made. The above description explains this level of performance.

The type of participant selection and the age range studied in this research made it possible to examine the maximum mental potential away from any age-dependent cognitive impairments or structural and functional immaturity of the brain. Identifying the cognitive mechanisms affecting conditional reasoning provides the knowledge of what basic cognitive skills should be developed to improve high-level cognitive skills. By using LISAS, we utilized a novel approach to integrate reaction times and accuracy scores, which were subsequently used to examine the variance. Using the WST version containing letters and numbers, we could eliminate the belief bias during the conditional reasoning test. However, we believe that adding the negation mode in addition to using the standard version reduces responding with biases and requires more thinking deeply [46]. So, the version combined with standard and negation rules is perfect for assessing the reasoning ability. Another limitation of the present study was the lack of investigation of education's effect on understanding the conditional rule. We suggest using a larger sample size and multifaceted cognitive batteries to assess inhibition and set-shifting in future studies that can reduce the effect of unique features of the tests and increase the validity of the results. Also, if different conditional reasoning tests are used, the effect of cognitive flexibility on conditional reasoning will be determined more precisely.

## CONCLUSION

To our knowledge, this is the first study that investigated the effect of inhibition and set-shifting on the rule-matching bias rate. In conclusion, we found evidence of the association between inhibition skill, set-shifting skill, and conditional reasoning. This result showed that both inhibition and set-shifting skills are essential for flexible thinking, updating target information, and conditional reasoning. The interplay between inhibition and set-shifting skills increases the logic-based reasoning ability and decreases the rule-matching bias rate.

## Personal thanks

All participants and experts from the National Brain Mapping Laboratory are appreciated.
